# Stereotactic Radiotherapy for Oligometastases in Lymph Nodes—A Review

**DOI:** 10.1177/1533033818803597

**Published:** 2018-10-23

**Authors:** Haruo Matsushita, Keiichi Jingu, Rei Umezawa, Takaya Yamamoto, Yojiro Ishikawa, Noriyoshi Takahashi, Yu Katagiri, Noriyuki Kadoya

**Affiliations:** 1 Department of Radiation Oncology, Tohoku University Graduate School of Medicine, Sendai, Japan

**Keywords:** oligometastases, stereotactic body radiation therapy, lymph node, metastases, gynecological cancer, breast cancer, prostate cancer, colorectal cancer

## Abstract

In recent years, the concept of oligometastases has become accepted and reports on stereotactic body radiotherapy as a treatment method have been published. Lesions in the brain, lung, and liver have been reported as target lesions. However, lymph node oligometastases could be a good candidate for stereotactic body radiotherapy as well. In this study, the usability of stereotactic body radiotherapy for oligometastases to lymph nodes is assessed by researching for each primary site. As a result, we could consider that stereotactic body radiotherapy could be almost well applied for lymph node oligometastases from the breast, gynecological organs, and prostate. However, doubts remain concerning the usefulness of stereotactic body radiotherapy for cervical node metastases from head and neck cancer or for mediastinal node metastases from lung or esophageal cancer since late toxicities have occurred with a large radiation dose at hypofractionation to major vessels or the central respiratory tract, especially in patients with irradiation histories. In addition, high-dose irradiation is required to control lymph node metastases from colorectal cancer due to its radioresistance, and severe late adverse events would therefore occur in adjacent organs such as the gastrointestinal tract. In cases of lymph node oligometastases with a primary tumor in the stomach or esophagus, stereotactic body radiotherapy should be used limitedly at present because this patient population is not so large and these metastases are often located close to organs at risk. Because of the varied status of recurrence and varied conditions of patients, it is difficult to determine the optimal dose for tumor control. It might be reasonable to determine the treatment dose individually based on dose constraints of adjacent organs. The oligometastatic state is becoming more frequently identified with more sensitive methods of detecting such oligometastases. In addition, there seems to be another type of oligometastases, so-called induced oligometastases, following successful systemic treatment. To determine the optimal indication of stereotactic body radiotherapy for lymph node oligometastases, further investigation about the mechanisms of oligometastases and further clinical studies including a phase III study are needed.

## Introduction

In 1995, the concept of an oligometastatic state was first proposed by Hellman and Weichselbaum as an intermediate state between a limited primary lesion and polymetastatic cancers.^1^ Interestingly, favorable outcomes have been achieved in patients with such oligometastases who received local ablative therapies. Oligometastatic state has been defined as a state with 5 or fewer clinically detectable metastases, though no definitive consensus exists.^2^ In 2010, Niibe and Hayakawa defined oligo-recurrence as a state with one to several distant recurrences with a controlled primary lesion.^3^ Patients with oligo-recurrence in a solitary region could be cured with local therapy such as stereotactic body radiotherapy (SBRT) with or without chemotherapy. Stereotactic body radiotherapy for oligo-recurrent metastases associated with the brain, lung, and liver has been considered to be a definitive treatment.

Results of some studies on SBRT for lymph node oligometastases were reported in 2009. Choi *et al* reported 30 patients with isolated para-aortic lymph node (PALN) metastases originating from uterine cervical and corpus cancer who had received SBRT using the CyberKife.^4^ Jereczek-Fossa *et al* reported 14 cases treated with SBRT for isolated lymph node recurrence from prostatic cancer.^5^ Kim *et al* reported 7 patients with isolated PALN recurrence from colorectal cancer treated with SBRT.^6^ They considered that SBRT for lymph node oligometastases has potential as salvage therapy. A review article about SBRT for lymph node oligo-recurrence by Jereczek-Fossa *et al* was published in 2015,^7^ and clinical guidelines for SBRT in lymph node oligometastases by the Spanish Society of Radiation Oncology (SEOR) were also published in 2015.^8^ The latest National Comprehensive Cancer Network (NCCN) guidelines include guidelines regarding SBRT for oligometastases.^9^ The description for cervical cancer in the guidelines is as follows: SBRT is an approach that allows delivery of very high doses of focused external beam radiation in 1 to 5 fractions and may be applied to an isolated metastatic site. Although there is no description in the guidelines for lymph node oligometastases, there is a description about SBRT as local therapy for oligo-recurrence in case of non-small cell lung cancer (NSCLC), rectal cancer, and colon cancer. Thus, worldwide attention has been given to SBRT as local therapy for lymph nodes.

The description in the SEOR guidelines is as follows: Although there is less experience in treating lymph node oligometastases with SBRT, several recently published series have shown that control rates higher than 60% were achieved with minimal toxicity in several diverse types of tumor including gynecological, prostate, colorectal, and gastric carcinoma and in diverse locations including the retroperitoneum, pelvis, upper abdomen, and groin.^8^ This review focuses on SBRT for lymph node oligometastases for each primary disease and revalidates the effectiveness, feasibility, and usability of SBRT.

## Gynecological Cancer

Oligometastases from gynecological malignancies have been considered as one of the most promising candidates for SBRT. The incidence of isolated PALN metastasis after definitive treatment for carcinoma of the uterus has been reported to be 1.7% to 12%.^10,11^ Matsuo *et al* reported that PALN recurrence was seen in 195 (4.2%) cases among 4663 clinically PALN-negative cases at surgery in a retrospective analysis of a Japanese nationwide cohort study of surgically treated stage IB–IIB cervical cancer.^12^

Although there have been some reports about SBRT for recurrent gynecological malignancies^13–15^ and some reports about SBRT for pelvic lymph node metastases from various primary diseases,^16^ as far as we know, there have been few review reports about particular cases of SBRT for recurrent lymph node metastasis from gynecological cancers. In 2009, Choi *et al* reported results of treatment for PALN metastases from uterine cancer.^4^ They treated 30 patients with isolated PALN metastasis from uterine cervical cancer (n = 28) and uterine corpus cancer (n = 2) using robot-assisted SBRT. However, the treatments were combined with chemotherapy and conventionally fractionated external beam radiotherapy depending on the situations, and the prescription doses ranged from 33 to 45 Gy in 3 fractions. The treatment response rate was 96% and the 4-year-treated tumor control and progression-free survival (PFS) rates were 67.4% and 45.0%, respectively. Grade 3 or more toxicity occurred in only 1 patient at 20 months after SBRT.

The results of a phase II clinical trial of robotic stereotactic body radiosurgery for metastatic gynecologic malignancies were reported by Kunos *et al* in 2012.^17^ Fifty patients were enrolled and underwent robot-assisted SBRT using 24 Gy in 3 fractions. The primary cancers in those patients were cervical cancer in 9 (18%) patients, endometrial cancer in 14 (28%) patients, ovarian cancer in 25 (50%) patients, and vulvar cancer in 2 (4%) patients. Stereotactic body radiotherapy sites were PALNs in 19 (38%) patients, pelvic lymph nodes/soft tissue in 14 (28%) patients, and other sites in 17 (34%) patients. Twenty-nine (58%) patients received SBRT as first-line therapy for metastatic gynecologic cancer disease. They reported that the SBRT target response rate, including the complete response (CR) and partial response rates, was 96% and that there was no treated tumor relapse in SBRT sites. However, distant metastases such as lung and liver metastases occurred in 31 (62%) patients. The incidence of SBRT-related toxicities of grade 3 or over, including noninfectious diarrhea, enterovaginal fistula, and hyperbilirubinemia, was 6%. In 2016, Hasan *et al* reported outcomes of SBRT for recurrent gynecological malignancies in 30 patients including 15 patients who received previous radiotherapy.^18^ The 5-year survival rate for 30 patients was 42% and the median survival period was 43.4 months with a median SBRT dose of 27.5 Gy (15-40 Gy) in 3 to 5 fractions. They reported 1 grade 3 toxicity (fistulation) and 2 grade 2 toxicities (rectal bleeding and hematuria). They described these cases in detail including the irradiated doses to critical organs.

As mentioned before, the description for cervical cancer in the NCCN guidelines is as follows: SBRT is an approach that allows delivery of very high doses of focused external beam radiation in 1 to 5 fractions and may be applied to an isolated metastatic site.^9^ Thus, lymph node oligometastases from gynecological cancer seem to be good indications for SBRT, though attention is needed for late toxicities in the gastrointestinal (GI) and urinary tracts.

## Prostate Cancer

Several review articles about SBRT for lymph node oligometastases from prostatic cancer were published in 2017. Those reports showed that SBRT is emerging as a treatment option for such oligometastases.

Ponti *et al* showed by a meta-analysis that treated tumor control was achieved in 98.1% of 211 patients who received SBRT for oligo-recurrent prostate cancer limited to lymph nodes.^19^ Jereczek-Fossa *et al* reported that in-field progression occurred in 9.7% of patients who received SBRT with a median dose of 24 Gy in 3 fractions for lymph node oligo-recurrent prostate cancer without grade 3 or 4 toxicity.^20^ Ingrosso *et al* reported that SBRT with a median dose of 35 Gy in 5 fractions resulted in a treated tumor control rate of 98% and that 40% of the patients experienced no disease recurrence with only 1 grade 3 GI toxicity.^21^ They demonstrated that SBRT is safe and effective. Ost *et al* reported patterns of failure after SBRT for oligo-recurrent nodal prostate cancer.^22^ They showed that most relapses (68%) occurred in nodal regions. Their data suggested that prophylactic whole pelvis irradiation might be effective. Ramey *et al* showed that adding whole pelvic radiation therapy had a potential to suppress relapse again.^23^ New diagnostic techniques such as 68Ga-labeled prostate-specific membrane antigen (68Ga-PSMA-11) positron emission/computed tomography (CT) may enable detection of micrometastases that have been difficult to find, and this may have an impact on the selection of treatment methods for salvage therapy including SBRT.^24^

The results of a prospective randomized multicenter phase II trial (Surveillance or metastasis-directed Therapy for OligoMetastasis Prostate cancer recurrence [STOMP]) for oligometastatic prostate cancer recurrence to assess the benefit of metastasis-directed therapy (MDT) were reported in 2017.^25^ In that study, 62 patients were enrolled and were randomly assigned to either surveillance or MDT (surgery or SBRT). Androgen deprivation therapy (ADT)-free survival was evaluated as the primary end point. The median ADT-free survival periods were 13 months for the surveillance group and 21 months for the MDT group with no severe toxicity of more than grade 3.

## Colorectal Cancer

Retroperitoneal recurrence was reported in 15% of colon cancer cases and 5% of rectal cancer cases.^26^ In the recent NCCN guidelines for resectable metachronous metastases of rectal cancer, it is stated that resection is preferred over locally ablative procedures (eg, image-guided ablation or SBRT); however, these local techniques can be considered for liver or lung oligometastases.^9^ In the NCCN guidelines concerning colon cancer, it is also stated that SBRT can be considered for oligometastases of the liver or lung, though there is no mention about lymph node oligometastases.

Curative surgical resection could be performed in only 6 of 38 patients with isolated retroperitoneal lymph node recurrence of colorectal cancer.^27^ Local treatment including SBRT might be meaningless. However, Kang *et al* reported that PFS rates were 25% at 3 years and 19% at 5 years in 31 patients with oligo-recurrence in pelvic lymph nodes from colorectal cancer treated by SBRT with a median dose of 42 Gy in 3 fractions.^28^ Bae *et al* reported that 2 patients treated with 48 and 51 Gy in 3 fractions among their 41 cases showed severe intestinal toxicities.^29^ Kim *et al* also reported that grade 4 intestinal obstruction occurred in 1 of 7 patients who received SBRT of 48 Gy in 3 fractions for PALN recurrence from colorectal cancer.^30^ Kang *et al* also reported that 2 (3%) of 41 cases had severe toxicities with 48 and 51 Gy in 3 fractions.^28^ Yeung *et al* reported that treatment doses were individualized based on location of tumor in relation to tolerance of nearby organs at risk and that coverage of the Planning Target Volume (PTV) was placed as lowest priority (below dose tolerance of organs at risk).^31^

In preoperative radiotherapy, Zlobec *et al* reported that a complete pathological response was about 6 times more likely in epidermal growth factor receptor (EGFR)-positive cases than in EGFR-negative cases.^32^ From this point of view, only patients with lower EGFR status may need a higher SBRT dose to achieve better treated tumor control. Lymph node oligometastases from colorectal cancer are mentioned as candidates for SBRT in SEOR, and favorable outcome can be expected.^8^

## Breast Cancer

Approximately 1% to 5.4% of patients with early-stage breast cancer have regional nodal recurrence after breast conservation treatment.^33–35^ Trovo *et al* reported relatively good results of a phase II study with SBRT or intensity-modulated radiation therapy for oligometastases from breast cancer.^36^ The main site of metastatic disease was the bone, but their cases included 23 cases of lymph node metastases that were treated without severe toxicity. Miyata *et al* reported that 19 (90%) patients with oligo-recurrence had an objective response to salvage radiotherapy and that the 3-year treated tumor control rate was 93%.^37^ There are few reports about SBRT for lymph node oligo-recurrence. However, according to available information on clinical experience, oligo-recurrent metastases of breast cancer seem to be good candidates for SBRT because the disease histories are generally not so aggressive. Stereotactic body radiotherapy for oligo-recurrence in regional lymph nodes from breast cancer in patients without a past irradiation history should be performed more proactively. On the other hand, because of the potential for prolonged survival of treated oligo-recurrent patients, late toxicities should be monitored carefully for a long period.

There is an ongoing research in this field: NRG-BR002 is an ongoing randomized phase II/III trial for assessing the role of SBRT or surgical ablation in patients with oligometastatic breast cancer.^38^

## Non-Small Cell Lung Cancer

Hishida *et al* reported that 162 (21%) patients had oligo-recurrence among 768 eligible patients with postoperative recurrences of lung cancer and that 73 of the 162 patients with oligo-recurrence had loco-regional recurrence.^39^ They also reported that their 3-year overall survival rate was 51.8%. Giuliani *et al* reported that 13.4% of 965 patients who received image-guided SBRT for early-stage NSCLC had regional recurrence.^40^ Thus, regional nodal oligo-recurrences after definitive treatments may be indications for SBRT in patients with NSCLC.

Gomez *et al* reported the results of a multi-institutional phase II randomized study on the local consolidative therapy (LCT) for oligometastatic NSCLC.^41^ In that study, the targets for consolidative therapy included the primary tumor, lymph nodes, and metastatic sites after front-line systemic therapy, and LCT included surgery, radiation therapy, or both. The trial stopped early after interim analysis because LCT extended PSF by 8 months (from 3.9 months in the observation arm to 11.9 months). Another interesting finding in the trial was that local therapy prolonged the time to appearance of a new lesion. It is possible that control of known lesions influences further metastatic spread, possibly by preventing further dissemination of known sites or by initiating a host response such as an immunologic reaction. Iyengar *et al* reported the results of a single-institution randomized phase II study of maintenance chemotherapy alone versus stereotactic ablative radiotherapy (SABR) followed by maintenance chemotherapy for patients with limited metastatic NSCLC.^42^ That trial was also terminated early. Progression-free survival in the SABR-plus-maintenance chemotherapy arm was 9.7 months and that in the maintenance chemotherapy-alone arm was 3.5 months.

Meng *et al* treated mediastinal lymph node metastases using SBRT.^43^ They used a median dose of 8 Gy/fraction and a median of 5 fractions and they evaluated the outcomes for 17 cases with prior radiotherapy compared with the outcomes for 16 cases without prior radiotherapy. The median overall survival period for patients without prior radiotherapy was 45.0 months, which was significantly longer than that (15.3 months) for patients with prior radiotherapy. Wang *et al* used SBRT for 85 patients with mediastinal lymph node oligometastases or oligo-recurrences from NSCLC.^44^ They reported that the 3- and 5-year overall survival rates after SBRT were 43.6%, and 21.3%, respectively, and that the overall survival of patients who received SBRT alone was significantly worse than that of patients who received SBRT with chemotherapy. They concluded that SBRT was safe except for patients who had a history of radiotherapy to mediastinal lymph node station 7. De Bari *et al* reported about patients who underwent reirradiation with SABR in 12 studies in terms of efficacy and toxicity in their review article.^45^ They concluded that thoracic reirradiation may offer satisfactory disease control; however, a particular caution should be paid in patients at high risk for radiation pneumonitis and, as central reirradiation carries substantial risk of high-grade toxicity, SABR should be probably limited to favorable disease presentations.

The recent NCCN guidelines include the following description for oligometastases of NSCLC: Definitive local therapy to isolated or limited metastatic sites (oligometastases; including but not limited to the brain, lung, and adrenal gland) achieves prolonged survival in a small population of well-selected patients with good performance status who have also received radical therapy to the intrathoracic disease. Definitive radiotherapy to oligometastases, particularly SABR, is an appropriate option in such cases if it can be delivered safely to the involved sites.^9^

## Gastric Cancer

Although gastric cancer is still one of the most common causes of death worldwide, cases of loco-regional recurrence alone are rare.^46^ Furthermore, Carboni *et al* reported that all of 6 patients in whom resection with curative intent was performed for postoperative isolated loco-regional recurrent gastric cancer died of recurrent gastric cancer, and they reported that surgery played a very limited role in treatment of isolated loco-regional recurrence of gastric adenocarcinoma.^47^ Nunobe *et al* reported that the 3- and 5-year survival rates were 36.7% and 9.8%, respectively, in patients in whom surgical treatment was performed for loco-regional recurrent gastric cancer.^48^

There are some reports about SBRT for oligo-recurrence from gastric cancer. Kim *et al* reported the efficacy of SBRT for PALN metastases after curative resection in patients with gastric cancer. They used 45 to 51 Gy in 3 fractions with robot-assisted SBRT, and the 3-year overall and PFS rates after SBRT were 43% and 29%, respectively, without any severe complications.^49^ Stereotactic body radiotherapy might be one of the options for curing postoperative recurrent gastric cancer. However, the role of SBRT for oligometastases in regional lymph nodes from gastric cancer is limited. In addition, in cases of SBRT for lymph node oligo-recurrence after curative resection, radiation planning is sometimes challenging because there are many surrounding normal tissues such as the liver, kidneys, adrenals, spinal cord, small intestine, and colon. National Comprehensive Cancer Network Guidelines v5.0. 2017 Gastric Cancer also do not recommend radiation therapy even for regional recurrence.^9^

## Head and Neck Cancer

Thariat *et al* carried out a cohort study of 880 patients with T1-4, N1-3 squamous cell carcinoma of the oropharynx, larynx, or hypopharynx treated with chemoradiation.^50^ They reported that 105 (12%) patients had nodal relapse and that the 5-year actual region control rate for patients with CR was 92%. Rivelli *et al* reported that there were 32 (11%) patients with lymph node metastases and 16 (6%) patients with both local recurrence and lymph node metastases in a follow-up study of 294 patients with treated oral squamous cell carcinoma.^51^

Thus, the incidence of lymph node oligo-recurrence of head and neck cancer is relatively high; however, there are few reports about SBRT for lymph node oligometastases in head and neck cancer. Kawaguchi *et al* reported 8 patients with lymph node metastases: 1 patient with a single retropharyngeal lymph node metastasis had CR, while the remaining 7 patients all progressed.^52^ In addition to insufficient treated tumor control, reirradiation sometimes results in severe adverse events (eg, carotid blowout). Therefore, the role of SBRT for lymph node oligometastases from head and neck cancer is considered to be limited at this time.

## Esophageal Cancer

Esophagectomy remains the standard treatment for patients with resectable esophageal cancer. Kyriazanos *et al* reported that 27% to 52% of patients who received surgery had recurrence and that loco-regional recurrence accounted for 41.5% to 55% of cases of postoperative recurrence.^53–58^ We reported that conventional chemoradiotherapy resulted in a 5-year overall survival rate of 39.2% in patients with solitary lymph node metastasis after curative surgery for esophageal cancer.^59^ Good results of radiotherapy for solitary lymph node metastasis after curative surgery for esophageal cancer were obtained; however, Yamashita *et al* showed by multivariate analysis that combined chemotherapy with radiotherapy was better than radiotherapy alone.^60^ To the best of our knowledge, there has been no report showing significant efficacy of SBRT in patients with lymph node oligo-recurrence of esophageal cancer, although there have been some studies that included a few patients with oligo-recurrence in lymph nodes from esophageal cancer.^31,61^ The reason might be that lymph node metastases to be treated exist close to critical organs including major vessels or the central respiratory tract.

## Stereotactic Body Radiotherapy Planning

The SEOR clinical guidelines for SBRT in lymph node oligometastases include recommendations about radiation planning for SBRT. In radiotherapy planning of SBRT for oligometastases, clinical target volume (CTV) is usually the same as the gross tumor volume. However, there are some reports about pathological findings concerned with extracapsular extension (ECE). From pathological examination of cases of lung cancer, Yuan *et al* showed that ECEs were <3 mm in 95% of the metastatic nodes, 3.2 mm in squamous cell carcinoma, and 3.5 mm in adenocarcinoma.^62^ Wang *et al* showed that the median ECE in metastatic lymph nodes of esophageal squamous cell carcinoma was 1.0 mm (range, 0.2-9.7 mm).^63^ They reported that ECE in 97.5% of metastatic lymph nodes was ≤5 mm. Ghadjar *et al* showed that ECE was ≤5 mm in 97% of patients with head and neck cancer.^64^

The ITV margin must be determined using 4D-CT, especially for abdominal lymph nodes. The mobility of adenopathies at the para-aortic level was estimated by Wysocka *et al* to be an average of 3.8 mm in craniocaudal displacement and less on other axes.^65^ Their results might support the setting of the CTV margin in SBRT planning.

Various fractionation schemes are described in the literature, which range between single doses of 24 Gy to schemes of 10 fractions of 5 Gy. While there is no clear scientific evidence for the dose threshold required to control metastases, we consider that the aim is to administer the highest dose that does not compromise the organs at risk.^8^

## Interpretation

Generally, SBRT for oligometastases in lymph nodes is relatively safe and effective, particularly in cases in which the primary cancer was breast, gynecological, or prostatic cancer. In these malignancies, the tumor-bearing survival time is relatively long and the frequency of oligo-recurrence is relatively high. In addition, it is important that the situation of organs at risk around the target is suitable for ablative SBRT. In contrast, the indication for SBRT is uncertain in cases of cervical lymph node metastases from head and neck cancer or in cases of mediastinal lymph node metastases from lung or esophageal cancer. In these cases, radiation therapy has been often used as the initial definitive cancer treatment, and the oligo-recurrence are often located very close to major vessels or the central respiratory tract. Therefore, attention must be given to the risk of severe late adverse events in major vessels or the central respiratory tract in radiation fields, especially in patients with irradiation histories.

On the other hand, high-dose irradiation has been considered to be required to control lymph node metastases from colorectal cancer due to its radioresistance. Because of a requirement of high-dose irradiation, there is a risk of severe late adverse events in peripheral normal organs such as the GI tract around the target. In cases of oligometastases to lymph nodes with a primary tumor in the stomach or esophagus, SBRT should be used limitedly at present because this patient population is not so large and these metastases are often located close to organs at risk.

Because of the varied status of recurrence and varied status of patients, it is difficult to determine unified optimal dose fractionations for tumor control. It might be reasonable to determine the treatment dose individually based on the tolerance dose of adjacent organs because it is possible to determine the dose constraint for each organ at risk. To determine the optimal indication of SBRT for oligometastases to lymph nodes, it is necessary to accumulate experiences and to analyze the outcomes.

There are few reports about patterns of failure following SBRT for lymph node oligometastases. Wang *et al* reported the patterns of failure in 85 patients with mediastinal lymph node oligometastases treated by SBRT.^44^ Among the 85 patients, 3 (4%) patients relapsed within the PTV and 49 (58%) patients had out-of-field progression. Among these 49 patients, 2 (2%) had diffuse progression including regional failure. Thirty-three (39%) patients had no progression after SBRT. Hasselle *et al* reported patterns of progression following hypofractionated image-guided radiotherapy to abdominal lymph node oligometastases.^66^ They treated 11 patients with 22 metastases. The primary sites were the kidney (n = 6), colon (n = 3), small bowel (n = 1), and endometrium (n = 1). Six (55%) patients progressed in new lymph nodes outside the PTV. Five (45%) patients progressed in adjacent lymph nodes as a component of the first failure. All adjacent failures occurred within 1 to 2 vertebral bodies of a treated lymph node. The 18-month actuarial lesion control rate was 74%. As mentioned before, Ost *et al* reported patterns of failure after SBRT for oligo-recurrent nodal prostate cancer. They showed that most relapses (68%) occurred in nodal regions.^22^ Their results may lead to advocate of an integrated boost technique.

Information about SBRT for lymph node oligometastases has been accumulating, but there is still limited information about treatment optimization and combination with new drugs, especially with molecular-targeted agents and immune checkpoint inhibitors.

The oligometastatic state is becoming more frequently identified with more sensitive methods of detecting such oligometastases. In addition, there seems to be another type of oligometastases, so-called induced oligometastases, following successful systemic treatment.^67^ Further investigation about the mechanisms of oligometastases and further clinical studies including a phase III study are needed.

## Abbreviations

ADT: androgen deprivation therapy
CTV: clinical target volume
CR: complete response
EGFR: epidermal growth factor receptor
ECE: extracapsular extension
GI: gastrointestinal
LCT: local consolidative therapy
MDT: metastasis-directed therapy
NCCN: National Comprehensive Cancer Network
NSCLC: non-small cell lung cancer
PALN: para-aortic lymph node
PFS: progression-free survivals
SBRT: stereotactic body radiotherapy
SEOR: Spanish Society of Radiation Oncology
SABR: stereotactic ablative radiotherapy.
